# Regional Inequities in Mammography Access and Utilization in Latin America: Ethnic, Rural, and Structural Barriers Identified Through a Narrative Review

**DOI:** 10.3390/epidemiologia7010025

**Published:** 2026-02-05

**Authors:** Nina Méndez-Domínguez, Mariana Jaqueline Arce Medina, Maricela Balam Gomez, Marco Esteban Morales Rojas, Esmeralda Novelo Moreno

**Affiliations:** 1 Hospital Regional de Alta Especialidad de la Peninsula de Yucatan, Servicios de Salud del Instituto Mexicano del Seguro Social, IMSS-BIENESTAR, Merida 97130, Mexico; 2 Faculty of Nursing, Universidad Autonoma de Yucatan, Merida 97000, Mexicomaricela.balam@correo.uady.mx (M.B.G.); a25214435@alumnos.uady.mx (E.N.M.)

**Keywords:** breast neoplasms, mammography, accessibility to health services, rural population, indigenous peoples

## Abstract

Background: Breast cancer remains a leading cause of morbidity and mortality among women in Latin America. Mammography is the most effective population-based tool for early detection; however, its impact is limited by persistent social, geographic, and structural inequities. Evidence from the region indicates that ethnicity, rural residence, and health system organization play a central role in shaping unequal access to screening services. Methods: We conducted a narrative review informed by a systematic search strategy, following PRISMA 2020 recommendations. Searches were performed in 17 international and regional databases in English and Spanish, covering publications from 2015 to 2025. Eligible studies included non-interventional quantitative designs reporting mammography access, utilization, or coverage among women residing in Latin American countries. Three reviewers independently screened records, extracted data, and classified determinants of inequality into sociodemographic, geographic, and health-system domains. Results: Of 532 records identified, 12 studies met the inclusion criteria, primarily from Mexico, Brazil, Peru, and Chile. Most analyses were based on nationally representative surveys. Mammography coverage ranged from approximately 20% to 60%, with consistently lower uptake among Indigenous women, rural populations, and women without health insurance. Reduced screening was associated with low educational attainment, socioeconomic disadvantages, rural residence, ethnic self-identification, and fragmented health system affiliation. Structural barriers, including concentration of diagnostic infrastructure in urban areas, reliance on opportunistic screening models, and limited capacity for systematic follow-up, were recurrent across countries. Conclusions: Inequities in mammography access and utilization in Latin America reflect deeply rooted social and structural determinants rather than a lack of screening technology alone. Reducing preventable breast cancer mortality requires strengthening organized, population-based screening programs, decentralizing diagnostic services, improving continuity of care, and implementing culturally appropriate strategies tailored to Indigenous, rural, and uninsured populations.

## 1. Introduction

Breast cancer is the most frequently diagnosed cancer and the leading cause of cancer-related mortality among women worldwide. In 2018, more than 2 million new cases and over 600,000 deaths were reported globally, with a growing burden in low- and middle-income countries (LMICs) [1,2]. In Latin America and the Caribbean, breast cancer incidence and mortality have increased steadily over the past two decades, reflecting demographic transitions, changes in reproductive patterns, and persistent inequalities in access to health services [3,4].

Mammography is internationally recognized as the most effective population-based screening tool for the early detection of breast cancer and the reduction in disease-specific mortality [5,6]. Even when mammographic sensitivity is lower in women under 50 years, randomized evidence shows that screening benefits and optimal intervals vary substantially by age, with reduced mortality impact in younger women, and in general, organized screening programs with adequate coverage, quality assurance, and timely diagnostic follow-up, mammography contributes substantially to early-stage diagnosis and improved survival [7,8]. However, in many LMICs, including most Latin American countries, screening remains largely opportunistic, fragmented, and dependent on individual initiative, which limits its public health impact [9,10].

The effectiveness of mammography is also influenced by age-related biological factors. As stated before, in women under 50 years of age, higher breast density reduces the sensitivity and specificity of mammography, increasing false-negative and false-positive results and limiting diagnostic accuracy [11]. These limitations underscore the need for age-appropriate screening strategies and complementary diagnostic approaches in younger populations, particularly in settings with constrained resources.

Despite international recommendations, access to mammography in Latin America remains highly unequal. Studies consistently report substantial socioeconomic gradients in screening participation, with lower utilization among women with limited education, informal employment, and lack of health insurance [9,12,13]. Health system fragmentation, characterized by parallel public and private subsystems, further reinforces these disparities by concentrating organized screening services among insured populations while leaving uninsured women dependent on irregular campaigns [14,15].

In Mexico, for example, although installed diagnostic capacity could theoretically support a broader screening coverage, empirical analyses show persistent underutilization and uneven geographic distribution of mammography services [16,17]. National studies indicate that effective coverage remains below 25% and is strongly concentrated in urban populations, despite the existing screening policies [18,19]. Similar patterns have been documented in Brazil, Peru, Chile, and Argentina, where screening infrastructures are often concentrated in metropolitan areas and higher-income regions [20,21,22,23].

Geographic and territorial barriers further limit access to screening. Rural and remote communities frequently lack nearby radiologic facilities, reliable transportation, and referral systems, resulting in delayed diagnosis and missed follow-up [23,24]. Spatial analyses in Brazil and Mexico demonstrate that mammography units are disproportionately located in economically developed regions, reinforcing existing social inequalities [22,24]. These territorial disparities intersect with socioeconomic vulnerability, intensifying exclusion among marginalized populations.

Ethnic and cultural factors also shape patterns of mammography use. Indigenous and Afro-descendant women in several Latin American countries exhibit lower screening participation, even after adjustment for socioeconomic status [25,26]. Language barriers, limited intercultural competences among health personnel, distrust of medical institutions, and the absence of female technicians could contribute to reduced engagement with screening services [26,27]. These mechanisms reflect broader processes of structural discrimination that become embodied through differential exposure to health risks and barriers to care [27,28].

At institutional level, weaknesses in referral systems, antique documentation management, and limited monitoring of screening performance undermine the continuity of care. Opportunistic screening models rarely ensure systematic follow-up of abnormal findings, leading to diagnostic delays and loss to follow-up, particularly among socially vulnerable women [29,30]. Consequently, increased availability of mammography equipment alone does not translate into improved population outcomes without integrated organizational structures.

Although several studies have examined determinants of mammography use in individual countries, a comprehensive synthesis of socioeconomic, territorial, and ethnic inequalities in screening across Latin America remains limited. Existing evidence is dispersed across disciplines and national contexts, making it difficult to identify shared structural patterns and policy-relevant mechanisms.

Therefore, this narrative review aims to synthesize current evidence on inequalities in mammography access and utilization in Latin America, with particular attention to socioeconomic, geographic, and ethnic determinants. By integrating findings from population-based, institutional, and spatial studies, this review seeks to contribute to a deeper understanding of how health system organization and social structures shape breast cancer screening outcomes in the region and to inform strategies for strengthening equitable early detection programs.

## 2. Materials and Methods

### 2.1. Study Design

**Figure 1:**
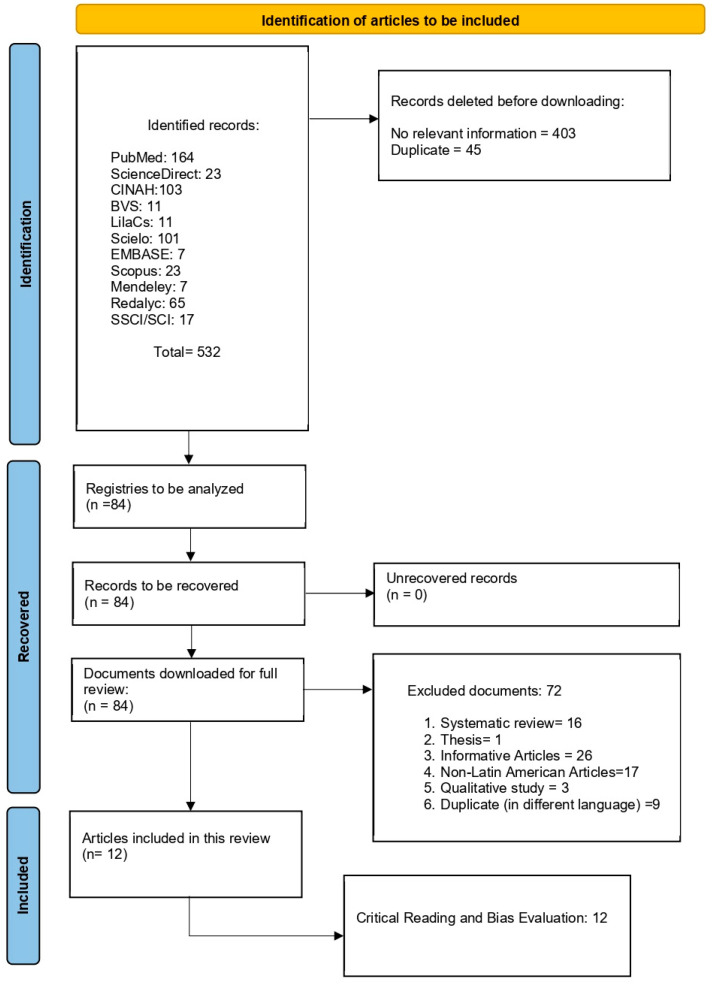
Flow chart of the information search process.

This study is a narrative review conducted with a systematic search strategy, aiming to synthesize evidence on inequalities in mammography access and utilization in Latin America. The review process followed the recommendations of the PRISMA 2020 flow diagram (Figure 1), and statement for transparency and reproducibility in evidence identification and selection (see also Table S1). Given the heterogeneity of study designs, data sources, and outcome definitions across the region, a narrative synthesis approach was adopted to integrate findings across countries and contexts. The review protocol was registered in the International Prospective Register of Systematic Reviews (PROSPERO; registration number CRD420251231287, 14 November 2025).

### 2.2. Research Team and Process

The review was conducted by a multidisciplinary team with training in epidemiology and public health. Three reviewers independently performed the literature search, screening, and data extraction. Discrepancies at any stage were resolved through discussion and consensus, with methodological supervision provided by senior investigators with experience in health systems research and evidence synthesis.

### 2.3. Search Strategy and Information Sources

A comprehensive search was conducted in 2025 across 17 international and regional databases, including ASSIA, CENTRAL, CINAHL, the Cochrane Library, Embase (via Ovid and Embase.com), LILACS, MEDLINE, PsycINFO, PubMed, Science Citation Index, Social Science Citation Index, and Scopus.

Search strategies were adapted to each database using controlled vocabulary (MeSH and DeCS terms) and free-text keywords in English and Spanish. Boolean operators were applied to combine terms related to breast cancer screening, mammography, access, coverage, inequity, and Latin America. An example of the core search string was:

(“breast cancer screening” OR “mammography” OR “mammogram”) AND

(“access” OR “coverage” OR “inequity” OR “inequality” OR “barriers” OR “socioeconomic” OR “rural”) AND (“Latin America” OR specific country names)

To maintain focus on original empirical evidence, records identified as systematic reviews, training programs, radiomics studies, or guideline-only publications were excluded at this stage.

### 2.4. Eligibility Criteria

Inclusion criteria were:

- Original, non-interventional quantitative studies
- Published between 2015 and 2025
- Written in English or Spanish
- Reporting mammography access, utilization, or coverage
- Conducted in populations residing in Latin American countries

Exclusion criteria were:

- Qualitative studies
- Interventional studies or clinical trials
- Editorials, commentaries, theses, conference abstracts without full text
- Studies not reporting results specific to Latin American populations
- Publications in languages other than English or Spanish

Studies conducted exclusively outside Latin America, including those focusing on migrant or diaspora populations (e.g., Mexican American or Indigenous American women residing in the United States), were excluded to preserve geographic and conceptual coherence

### 2.5. Study Selection Process

The selection process followed three sequential phases:

1. Identification: A total of 532 records were retrieved across all databases.
2. Screening: After removal of 45 duplicate records, titles and abstracts of the remaining records were screened independently by three reviewers.
3. Eligibility: Full-text assessment was conducted for 84 articles. Of these, 72 were excluded due to ineligible study design, population, or outcome focus.

Ultimately, 12 studies met all inclusion criteria and were included in the narrative synthesis. The full selection process is detailed in the PRISMA 2020 flow diagram, ensuring traceability of inclusion and exclusion decisions.

### 2.6. Data Extraction and Synthesis

For each included study, data were extracted using a standardized form capturing country, year of publication, study design, data source, population characteristics, mammography coverage or utilization estimates, determinants examined and reported limitations or biases. Determinants of inequality were grouped into three analytic domains:

1. Socio-demographic (e.g., education, income, ethnicity)
2. Geographic (e.g., rural residence, regional availability)
3. Health system-related (e.g., insurance affiliation, program organization) Given heterogeneity in outcome definitions and analytic approaches, quantitative pooling was not performed. Instead, findings were synthesized narratively, emphasizing consistency and divergence across countries.

### 2.7. Methodological Quality and Risk of Bias

The methodological quality of included studies was assessed using domains adapted from the Joanna Briggs Institute tools for observational studies, including selection bias, measurement validity, control of confounding, and analytic rigor. Each study was independently classified as having low, moderate, or high risk of bias by two reviewers. Disagreements were resolved by consensus. Risk-of-bias assessments were used to contextualize findings rather than to exclude studies from the synthesis.

## 3. Results

A total of 12 studies met the inclusion criteria and were included in the narrative synthesis. The included studies provided evidence exclusively from Latin American countries, primarily Mexico, Brazil, Peru, and Chile. Most studies were based on nationally representative population surveys, while a smaller number used ecological, spatial, or administrative datasets.

The predominant study design was analytical cross-sectional, although some studies employed ecological or spatial analyses to assess regional availability of mammography services. Study periods ranged from the early 2000s to 2022, depending on the data source. All studies focused on women within nationally defined target age groups for breast cancer screening; ecological analyses from Brazil and Mexico provided complementary evidence on geographic and socioeconomic gradients in screening availability.

### 3.1. Mammography Access and Coverage

Consistent inequalities in mammography utilization were documented across all included countries. In Chile, Guerrero-Nancuante et al. reported that women in the lowest income quintiles were almost 50% less likely to undergo mammography, despite the exam being offered free of charge within the public health system [21]. In Peru, Hernández-Vásquez [31], Alegría-Delgado, found that national mammography coverage remained low (23–25%), with rates dropping below 15% among rural and uninsured women, reflecting structural and educational barriers to access [32].

In Mexico, Alvarado-López et al. documented an effective mammography coverage rate of 19.7%, noting that screening services were largely concentrated among urban residents and women affiliated with social security institutions [17]. Using longitudinal survey data, McClellan et al. observed an increase in mammography utilization from 14% to 24% between 2001 and 2018; however, substantial disparities persisted across socioeconomic groups [18]. An economic simulation conducted by Ulloa-Pérez et al. estimated that expanding screening coverage to 70% could reduce breast cancer mortality by up to 20% under modeled scenarios [33].

In Brazil, Nogueira et al. reported that women with lower educational attainment and those residing in the northern and northeastern regions had significantly lower probabilities of undergoing mammography compared with women living in the more developed southeastern region of the country [22].

### 3.2. Geographic Determinants and Rurality

Geographic inequalities in mammography access were consistently reported across the included studies. In Brazil, Bezerra et al. demonstrated that rural regions had fewer than one mammography unit per 100,000 women, and that higher levels of social vulnerability were associated with lower screening rates [24]. In Peru, Alegría-Delgado reported that women residing in rural areas were approximately three times less likely to undergo mammography than urban women, even after adjustment for educational level and insurance status [32]. Studies from Mexico have reported low screening adherence, limited coverage, and the need for strengthened community-based screening practices, with a substantial proportion of women never having undergone mammography or clinical breast examination which also indicates variable local program limitations [34,35].

### 3.3. Ethnicity and Sociocultural Determinants

Ethnicity was consistently identified as a determinant of inequality in mammography utilization. In Chile, Guerrero-Nancuante et al. documented lower participation in mammography screening among Indigenous women compared with non-Indigenous women [21]. In Brazil, Nogueira et al. reported substantially lower screening uptake among Afro-descendant women, even after adjustment for socioeconomic conditions [22]. In Mexico, Alvarado-López et al. found that Indigenous self-identification was associated with a decreased likelihood of undergoing mammography, an effect partially mediated by educational attainment and type of health insurance affiliation [17].

An external evaluation of the national mammography screening program in Mexico reported an effective coverage of approximately 19% in 2017, with major deficiencies in diagnostic follow-up and referral systems [36], while Câmara et al. identified socioeconomic and organizational factors as major contributors to barriers to breast cancer screening in Brazil [37].

Across the included studies, average mammography coverage ranged between 20% and 60%, with lower levels consistently observed among rural populations, Indigenous groups, and uninsured women. The most frequently reported determinants of reduced screening included lower educational level, lower socioeconomic quintile, type of health system affiliation, and geographic location. Recurrent sources of bias and methodological limitations are summarized in Table 1. Common methodological limitations reported across studies included reliance on self-reported mammography use, under-representation of rural areas, and ecological bias [22,24,38].

Quality and potential bias. Studies based on nationally representative surveys (Chile CASEN; Peru ENDES; Mexico ENSANUT; Brazil PNS) generally exhibited low to moderate risk of bias, primarily due to reliance on self-reported mammography use and the possibility of residual confounding. In contrast, spatial ecological studies and analyses of administrative records (including Brazilian micro-regions, Brazilian municipalities, and state-level screening programs in Chihuahua and Jalisco) demonstrated moderate to high risk of bias, particularly related to under-reporting and the absence of individual-level adjustment.

The economic simulation study and the review of breast cancer screening in developing countries were classified as high risk of bias when considered as empirical evidence. Consequently, these studies were used exclusively to describe cost-effectiveness assumptions and organizational characteristics of screening systems rather than to estimate population-level coverage or utilization outcomes.

## 4. Discussion

The findings of the present review consistently indicate that socioeconomic, territorial, and ethnic inequalities are key determinants of low mammography coverage in Latin America. Previous evidence from Latin America indicates that inequalities in early breast cancer detection are driven by socioeconomic and institutional conditions rather than by infrastructure alone [38,39], summing to evidence suggesting that screening programs in developing countries are predominantly opportunistic, with limited infrastructure and low population coverage, contributing to late-stage diagnosis [40]. This is congruent with the findings from a national external assessment revealing persistent weaknesses in program monitoring, quality assurance, and continuity of care [41]. Program implementation and monitoring limitations along with structural barriers limit their effective use, while also, limited knowledge, fear of diagnosis, and misinformation remain major barriers to screening participation.

Based on the synthesized evidence, determinants of inequality in mammography coverage can be conceptualized across three interdependent levels: (a) structural, reflecting broader social and economic inequalities; (b) institutional, related to the organization and performance of health services and infrastructure; and (c) territorial, associated with geographic location and cultural accessibility. Studies by McClellan et al., Câmara et al., and Balandrán-Duarte et al. demonstrate that gaps in early detection do not primarily result from a lack of technology, but rather from the interaction of social position, labor conditions, insurance affiliation, and ethnic–territorial factors that determine who can effectively access mammography services and who remains excluded [18,39]. This interpretation aligns with Breilh’s perspective that health and disease processes are shaped by the social structures that produce them, and that inequalities in screening access reflect broader “social determinations of life” that perpetuate inequity [40].

The fragmentation of Latin American health systems, characterized by parallel insurance schemes, further reinforces internal inequities [23,41]. Contributory insurance systems tend to provide organized screening with defined referral pathways, whereas public subsystems frequently rely on opportunistic campaigns lacking systematic follow-up.

In Brazil, recent efforts to transition toward more organized screening programs seek to address these institutional gaps, particularly in the context of racial inequities, as Black women experience a substantially higher risk of breast cancer mortality compared with white women [32]. These patterns are consistent with Krieger’s ecosocial framework, which emphasizes how structural discrimination becomes biologically embodied through cumulative disadvantage over the life course [27,28].

Geographic access also plays a decisive role in determining who benefits from mammography screening. Diagnostic equipment is disproportionately concentrated in metropolitan areas where higher-income, more educated, and insured populations reside [26]. In Peru and Mexico, long distances to radiologic centers and limited public transportation represent significant barriers to screening participation [34]. In Brazil, approximately 70% of mammography machines are in the southeast region, leaving the Amazon and northeastern regions underserved [38]. These territorial inequalities exacerbate the vulnerability of rural women, who face the combined effects of poverty, geographic isolation, and limited continuity of care.

Beyond material barriers, symbolic and cultural dimensions influence screening behaviors. Among Indigenous and Afro-descendant communities, mammography may be associated with fear, shame, or distrust toward health personnel [22]. The lack of female technicians and intercultural mediators can further distance communities from services, reinforcing perceptions that mammography is intended for “other women.” These experiences illustrate the material consequences of racism and historically rooted forms of exclusion within health systems.

### Who Is Mammography for and Who Is It Not For?

Across the region, the evidence suggests that mammography in Latin America is primarily accessed by urban, insured, and more educated women with stable employment, who are better positioned to navigate health systems, travel to diagnostic centers, and receive timely follow-up. In contrast, rural, Indigenous, and uninsured women face structural, geographic, cultural, and economic barriers that substantially limit their access to screening.

The challenges observed in Latin America are not unique; evidence from countries with diverse income levels but fragmented health systems such as the United States, India, and South Africa, among others, demonstrates that breast cancer screening and care are frequently shaped by insurance status, urban concentration of diagnostic infrastructure, and public–private segmentation. These structural features consistently translate into delayed diagnosis, disrupted care pathways, and disproportionate barriers for uninsured, low-income, and rural women, underscoring that inequitable access to mammography is a global manifestation of health system fragmentation rather than a region-specific limitation.

In practice, access to mammography may function as a marker of health citizenship, distinguishing women who are recognized and served by health systems from those who remain marginalized. Addressing this gap requires redistributive policies, diagnostic decentralization, and systematic monitoring of class-based and racialized inequities as central social determinants of health [5,6,15,40]. Finally, proactive identification of eligible women, community-based education, systematic monitoring of participation rates, standardized recall systems, timely diagnostic assessment, defined diagnostic intervals, and strengthened information systems is desirable to ensure continuity of care [42].

## 5. Conclusions

Only when breast cancer early detection strategies are articulated through principles of social justice, cultural relevance, and an understanding of the lived realities of diverse populations can mammography be considered a truly equitable public health intervention. Moving beyond technocratic approaches to screening requires recognizing how structural inequities shape who can access mammography and who ultimately benefits from early detection.

Ensuring that mammography is accessible “for all” women in Latin America implies redesigning screening programs with community participation, strengthening primary care referral pathways, reducing financial and logistical barriers, and integrating intercultural models of care that respect local knowledge, cultural contexts, and women’s preferences. Aligning screening technologies with equity-oriented policies and context-sensitive implementation is essential for mammography to fulfill its potential as a population-level tool for reducing breast cancer morbidity and mortality in the region.

## Figures and Tables

**Table 1:** Characteristics of the 12 articles included in the present review regarding mammography in Latin America population.

First Author Lastname	Country	Year	Main Result	Determinants Identified	Reported Biases or Limitations
Guerrero-Nancuante [21]	Chile	2023	58% coverage in urban areas vs. 31% in rural areas	Socioeconomic status, education, health insurance	Self-reported data; potential cultural misclassification
Hernández-Vásquez [31]	Peru	2019	23% national coverage; 13% in rural areas	Education, wealth quintile, region	Self-report; limited adjustment for geographic availability
Alegría-Delgado [32]	Peru	2017	25% urban coverage vs. 9% rural	Area of residence, insurance status, educational level	Incomplete data; selection bias
Alvarado-López [17]	Mexico	2020	19.7% effective mammography coverage	Education, insurance affiliation, income quintile	Self-report; regional variability
McClellan [18]	Mexico	2023	Increase from 14% to 24% (2001–2018)	Insurance type, age, socioeconomic level	No cluster adjustment; non-response bias
Ulloa-Pérez [33]	Mexico	2016	Modeled scenario: 70% coverage associated with 20% mortality reduction	Cost-effectiveness assumptions, age scenarios	Homogeneous assumptions; parameter uncertainty
Nogueira [22]	Brazil	2020	60% coverage in urban areas vs. 36% in rural areas	Income, schooling, region, insurance	Under-registration in rural areas; regional variability
Bezerra [24]	Brazil	2022	Density < 1 mammography unit per 100,000 women	Social vulnerability, rurality	Ecological bias; variable data quality
Aguilar-Torres [35]	Mexico	2021	Average coverage of 30%	Program organization, service availability	Incomplete data; lack of national representativeness
García Romero [34]	Mexico	2022	Average coverage of 25%	Local campaigns, institutional coordination	Lack of validation; information bias
Romo-Dueñas [36]	Mexico	2025	External evaluation showed low coverage (~19%) and major gaps in follow-up and diagnostic capacity	Program capacity, organization, referral systems, regional marginalization	Program evaluation design; administrative data limitations
Câmara [37]	Brazil	2025	Sociodemographic factors associated with barriers to breast cancer screening	Education, income, access to information, insurance status	Cross-sectional design; self-reported barriers

## Data Availability

Available as supplementary material and at ResearchGate profiles of corresponding authors.

## Supplementary Materials

The following supporting information can be downloaded at: https://www.mdpi.com/article/10.3390/epidemiologia7010025/s1. Table S1: Prisma Checklist table [43].
